# Mobile Technology Use in Clinical Research Examining Challenges and Implications for Health Promotion in South Africa: Mixed Methods Study

**DOI:** 10.2196/48144

**Published:** 2024-04-08

**Authors:** Khuthala Mabetha, Larske M Soepnel, Gugulethu Mabena, Molebogeng Motlhatlhedi, Lukhanyo Nyati, Shane A Norris, Catherine E Draper

**Affiliations:** 1 South African Medical Research Council/Wits Developmental Pathways for Health Research Unit, Department of Paediatrics, Faculty of Health Sciences, School of Clinical Medicine University of the Witwatersrand Johannesburg South Africa; 2 Julius Center for Health Sciences and Primary Care, University Medical Center Utrecht University Utrecht Netherlands; 3 School of Human Development and Health University of Southampton Southampton United Kingdom

**Keywords:** mobile technologies, health promotion, mixed methods, clinical practice, mobile phone

## Abstract

**Background:**

The use of mobile technologies in fostering health promotion and healthy behaviors is becoming an increasingly common phenomenon in global health programs. Although mobile technologies have been effective in health promotion initiatives and follow-up research in higher-income countries and concerns have been raised within clinical practice and research in low- and middle-income settings, there is a lack of literature that has qualitatively explored the challenges that participants experience in terms of being contactable through mobile technologies.

**Objective:**

This study aims to explore the challenges that participants experience in terms of being contactable through mobile technologies in a trial conducted in Soweto, South Africa.

**Methods:**

A convergent parallel mixed methods research design was used. In the quantitative phase, 363 young women in the age cohorts 18 to 28 years were contacted telephonically between August 2019 and January 2022 to have a session delivered to them or to be booked for a session. Call attempts initiated by the study team were restricted to only 1 call attempt, and participants who were reached at the first call attempt were classified as contactable (189/363, 52.1%), whereas those whom the study team failed to contact were classified as hard to reach (174/363, 47.9%). Two outcomes of interest in the quantitative phase were “contactability of the participants” and “participants’ mobile number changes,” and these outcomes were analyzed at a univariate and bivariate level using descriptive statistics and a 2-way contingency table. In the qualitative phase, a subsample of young women (20 who were part of the trial for ≥12 months) participated in in-depth interviews and were recruited using a convenience sampling method. A reflexive thematic analysis approach was used to analyze the data using MAXQDA software (version 20; VERBI GmbH).

**Results:**

Of the 363 trial participants, 174 (47.9%) were hard to reach telephonically, whereas approximately 189 (52.1%) were easy to reach telephonically. Most participants (133/243, 54.7%) who were contactable did not change their mobile number. The highest percentage of mobile number changes was observed among participants who were hard to reach, with three-quarters of the participants (12/16, 75%) being reported to have changed their mobile number ≥2 times. Eight themes were generated following the analysis of the transcripts, which provided an in-depth account of the reasons why some participants were hard to reach. These included mobile technical issues, coverage issues, lack of ownership of personal cell phones, and unregistered number.

**Conclusions:**

Remote data collection remains an important tool in public health research. It could, thus, serve as a hugely beneficial mechanism in connecting with participants while actively leveraging the established relationships with participants or community-based organizations to deliver health promotion and practice.

## Introduction

The use of mobile technologies in engaging people and communities to choose healthy behaviors and to improve their health is becoming an increasingly common phenomenon in global health programs [1-4]. Mobile technologies have increasingly become a common mode of delivering telemedicine in sub-Saharan Africa and other low- and middle-income countries (LMICs) given improvements in coverage [5], with the prospects of improving access to information pertaining to health care services in areas where health care systems lag in meeting minimum health standards [6,7]. Mobile phone technology use in clinical studies is a potential avenue for both the follow-up of participants in research [8-10] and the provision of targeted and tailored messaging to facilitate behavior change support. For instance, patients now progressively receive reminders regarding clinic appointments [11], medication uptake [12], health promotion [13,14], medical treatment or diagnosis [15], and accessing information on disease outbreaks and other health-related data [16] through SMS text messaging systems and telephone calls; all these methods have collectively contributed to better health [1,17]. These digital interventions are reinforced by the high level of mobile phone penetration in many LMICs.

Mobile technologies typically exist in the form of internet-enabled devices such as smartphones, tablets, and watches [18]. Mobile phones constitute the most commonly used mobile technologies for cellular communication in LMICs [19,20] and are used as a cellular communication system for making and receiving phone calls, video calls, text and instant messaging, surfing the internet, GPS navigation, and playing games [21]. For the purposes of this study, the focus was particularly restricted to mobile phones, with the form of cellular communication being restricted only to phone calls.

Research conducted in the sub-Saharan African region has shown that individuals who participate in research studies are intermittently reached through the phone, which is the most commonly used method in the region [22]. Among African countries, South Africa has the third highest number of people who are active mobile phone users, providing a robust platform for telemedicine prospects [22]. For example, 97% of households in South Africa have access to a mobile phone [23]. According to the Independent Communications Authority of South Africa 2019 report on the state of information and communications technology, an estimated 82% of the country’s total population have a smartphone [24], with 88% of the population reported to have prepaid mobile cellular subscriptions and 12% of the population reported to have contract subscriptions [24]. Although mobile technologies have shown some success in their effectiveness in health promotion initiatives and in improving health knowledge, the use of mobile phones in delivering these interventions within clinical practice and research raises some concerns [25]. For example, although a large proportion of people own mobile phones, there are low retention rates in clinical research, and this has been largely attributed to challenges of participant reachability and accessibility by phone [22]. For instance, in a clinical trial conducted in Togo, out of an approximate proportion of 13,726 respondents who had provided their mobile number, 80% could not be reached on that number [26].

In addition, research conducted in Côte d’Ivoire among patients who were receiving antiretroviral treatment showed that out of the 7000 patients who were traced telephonically after initially actively participating in the research but were lost at the point of follow-up in the study, only 40% of the patients could be reached on the telephone number that was provided [27]. A randomized controlled trial conducted in South Africa among 400 HIV-positive patients found that only 60.3% of the patients could be reached telephonically after being contacted a maximum of 3 times via telephone on different days and at different times for every phone number that the participant had provided [22]. In addition, another South African study indicated that reachability by phone is a common challenge in the country. This is caused, for instance, by theft and loss of phones, which results in connectivity loss with respondents and loss to follow-up [28]. Although several scholars have investigated the effectiveness of mobile phones in follow-up studies [1,6-8,13,24,27-29], there is a dearth of literature in South Africa that has qualitatively explored the challenges that participants experience in terms of being contactable through mobile technologies. Gaining insight into these challenges will help in developing targeted strategies to facilitate ways for research staff and researchers to remain in contact with participants over time and for follow-up research. The results could also potentially inform the use of mobile technologies for reaching patients in a clinical setting.

The trial is being conducted in Soweto, South Africa. It is the *Bukhali* preconception health trial, which is nested in the Healthy Life Trajectories Initiative [30]. The aim of the trial is to optimize the physical and mental health of young women, fostering positive dynamic changes in their health for themselves, and where relevant, for future cohorts. In this trial, young women received intervention materials and resources delivered by community health workers, referred to as “Health Helpers.” These materials include health literacy materials, multimicronutrient supplements, health screening and referral conducted in person, nutritional risk and support, and monthly sessions that help with modifying or transforming health behaviors. The monthly sessions were conducted telephonically by the Health Helpers with on-site visits scheduled every 6 months [30-32]. However, there have been challenges in contacting participants for booking and delivering monthly trial sessions because the participants were unreachable on the contact numbers registered for the study. Therefore, this study aimed to explore the challenges that participants experienced with regard to being contactable through mobile technologies, using both quantitative and qualitative data.

## Methods

### Study Design

A convergent parallel mixed methods research design was applied. It is a research design that involves the collection and analysis of quantitative and qualitative data separately, which are then compared simultaneously to better understand the phenomenon under study [33]. The main presumption of the research design was that both quantitative and qualitative data offer varying information, with the qualitative data providing narrative accounts of the participants and the quantitative data providing scores on selected variables [33]. This design provides a broader insight into the issue being studied by comparing the findings and results to discern whether they confirm or disconfirm each other. Combining both qualitative and quantitative methods as complementary methods has the potential to contribute to broader applicability of the findings in other settings or with other wider populations [33-36]. This method is applicable in this study given that the quantitative data provided insight into data on many Bukhali trial participants, whereas the qualitative data provided an in-depth narrative account of the mobile phone challenges faced by participants and other reasons for being hard to reach.

### Participants and Recruitment

The study population consisted of young women aged 18 to 28 years who participated in the trial within the precinct of the Chris Hani Baragwanath Academic Hospital, located in Soweto, Johannesburg, South Africa, and were recruited into the intervention arm of the *Bukhali* trial [30]. Soweto is a largely overpopulated and multilingual area that lies on the outskirts of the greater Johannesburg city. It is characterized by several socioeconomic issues, namely, joblessness, gender-based violence, and food insecurity, as well as factors hindering healthy behaviors [37], which have the potential to influence the mobile phone use patterns of young women.

Overall, the study team contacted participants telephonically to (1) deliver monthly sessions to the participants, (2) book subsequent monthly sessions for the participants, (3) arrange to have supplements delivered to the participants’ homes, or (4) follow-up on the participants who have experienced an adverse event. The mobile phone information of the participants was captured by the study team to enable the team to reach the participants. For the quantitative component of this study, we included all young women who were contacted telephonically between August 2019 and January 2022. These women were contacted to either have a session delivered to them or to be booked for a session (n=363) out of the wider group of Healthy Life Trajectories Initiative participants. This was done to identify young women who were reachable and those who were unreachable. Participants who had (1) in-person contact with the study team (either off-site to deliver supplements or on-site to deliver a session), (2) contact via SMS, or (3) contacted through other means of communication (eg, home visits or instant messaging) were excluded from this study to focus on the extent to which participants could be contacted by phone.

**Figure 1:**
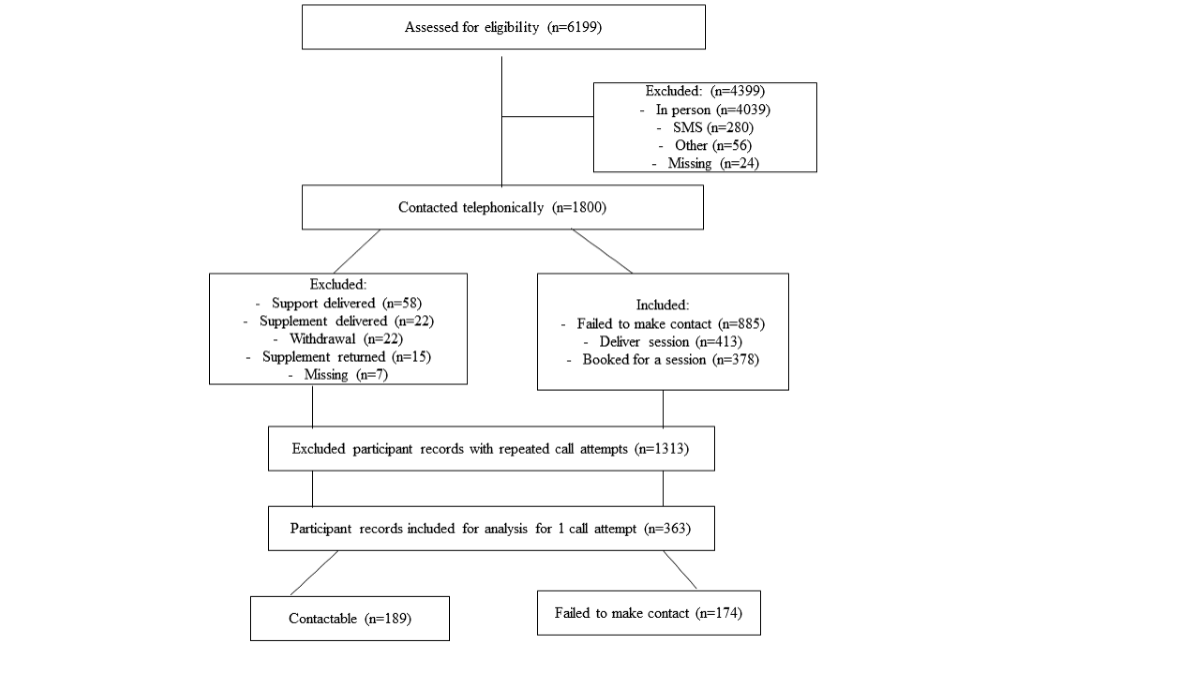
Flow diagram of the trial participants contacted telephonically between August 2019 and January 2022.

The standard process used by the intervention team to facilitate the intervention involved making several call attempts to reach the participants. All call attempts and contact statuses of the participants were captured on a contact log. At most, 3 call attempts were made and if the participants were still unreachable, the study team generated tracing lists that prompted them to trace these participants by conducting further fieldwork to update the contact details of the participants. For the purposes of this study, we restricted the call attempts to only 1 call attempt given that the study only aimed to provide a cross-sectional picture and not an analysis that focuses on more longitudinal repeated attempts, which was beyond the scope of this study. Thus, participants who were reached at the first call attempt were classified as “reachable.” Participants whom the study team failed to make contact with at the first call attempt were classified as “hard to reach” or “failed to make contact.” In the same period, some participants changed their mobile number once, some did it several times, and others did not change their number. A flowchart indicating the stages of participant recruitment is shown in Figure 1.

For the qualitative component, a subsample of participants (n=20) who were part of the *Bukhali* trial for ≥12 months were recruited telephonically for those who were easily contactable, and recruitment was performed by a fieldwork team for those who were hard to reach. This sample of participants was purposively selected using a convenience sampling technique. A group of 20 participants was selected by drawing them from the same group of young women who participated in the trial between August 2019 and January 2022. The final group of women who participated in the trial would be booked to receive a monthly session either in person, telephonically, or through SMS or to have supplements delivered to them. We then first restricted this group to only young women whom we failed to make contact with, those who had a session delivered to them, and those who were booked for a session. Furthermore, participants who could not be reached after repeated call attempts were excluded, and we then further restricted the sample to those who only had 1 call attempt. This resulted in a group of those who were contactable and those who were hard to reach, and this group was included in the quantitative analysis. Thereafter, 20 young women who were included in the qualitative analysis were selected from the 363 young women who were included in the quantitative analysis. This was done by selecting the record IDs of young women extracted from REDCap (Research Electronic Data Capture; Vanderbilt University), a secure database that stores data for research studies [38]. Qualitative data were collected only for this group of young women given that the trial aimed to deliver an intervention that fosters behavior change. Thus, the lack of contactability or loss to follow-up of this group of participants poses a substantial threat to the effective delivery of the intervention and could potentially have adverse biases on the conclusiveness of the results of the trial, which could have significant effects on the credibility and reliability of the trial.

### Data Collection

#### Quantitative Data Collection

Quantitative data collection was conducted, and the data were recorded and stored on REDCap [38]. The data included a set of interview-led administered questionnaires that collected baseline data on the demographic characteristics of the participants. The variables that were used in this study to show a descriptive profile of the sociodemographic characteristics of the participants who were contactable and those who were hard to reach included (1) age, (2) family size, (3) highest level of education, (4) vocational activity, (5) type of dwelling unit, (6) times in the past 12 months when family went hungry, (7) phone ownership, (8) contract type, and (9) type of cell phone.

In this component of the study, the first outcome of interest was the “contactability” of the participants. Two groups of young women were of interest in this phase of analysis. These were (1) young women who were contactable and (2) young women who were hard to reach at the first call attempt. The second outcome was the “mobile number changes of participants,” which were captured on REDCap. These number changes were tracked from randomization to the trial. The number changes were categorized as (1) no number change, (2) 1 number change, or (3) >2 number changes.

#### Qualitative Data Collection

Overall, 20 participants were interviewed individually using an in-depth interview guide by 2 female interviewers who were fluent in both English and other South African vernacular languages. The interview schedule was developed by the study team to capture the participants’ reasons for changing their mobile numbers and to discern why staying in contact with participants was challenging. Interview questions focused on young women’s demographic characteristics, questions around ownership of a mobile phone or mobile phones, mobile phone use behavior and patterns, and receipt of health information. The interviews were conducted face-to-face with the participants within the precinct of the Chris Hani Baragwanath Academic Hospital in February and March 2022. The interviews lasted between 30 and 40 minutes and were audio recorded. Interview notes were captured during each scheduled interview session to record the participants’ main accounts and nonverbal cues. Before analysis was conducted, the recordings were transcribed to their original form and translated into English, where necessary.

### Data Analysis

#### Quantitative Data Analysis

To statistically analyze the differences between the 2 groups of participants (failed to make contact and contactable), the data were analyzed descriptively at univariate and bivariate levels. At the univariate level, to visually illustrate the differences between the contact status of participants by mobile number change, a graph was generated to compare the proportion or rate of mobile number change of each group in relation to another. Similarly, another graph was generated to identify the most cited and least cited outcomes of the attempted call for participants we failed to make contact with. At the bivariate level, a contingency table was generated to depict the differences in the results of the tabulation of observations at each level of a variable for both groups, that is, cross-tabulation of the 2 groups to show the relationship or differences in the proportions and frequencies of the sociodemographic characteristics of the 2 groups. This table included the Pearson chi-square test of association to statistically identify the independence or association between the 2 groups or categorical variables and accompanying sociodemographic covariates. All data were analyzed using STATA (version 17; StataCorp) [39].

#### Qualitative Data Analysis

A reflexive thematic analysis approach was used for analysis using MAXQDA software (version 20; VERBI GmbH) [36], which helped in the recording, coding, and interpretation of the transcripts. Reflexive thematic analysis is an interpretive method that enables researchers to establish the outcome of the work rather than following a specific theory [35]. Six steps were used in the analysis. In the first step, data familiarization was conducted by checking the quality of the transcripts against the recordings to understand the data thoroughly, which would make it possible to search for patterns and meanings. This involved reading and rereading transcripts and taking notes. In the second step, initial codes were generated by labeling and organizing participants’ narratives to form a complete meaning.

In the third phase, initial themes were generated by sorting initial codes that had been generated into themes and identifying meanings and relationships between the initial codes. In the fourth phase, the themes were reviewed by identifying coherent patterns and ensuring that the generation of themes was supported by sufficient data. This also included collapsing overlapping themes and regenerating and improving the codes and themes. This was done as a collective effort among KM, LMS, and CED. In the fifth phase, themes were defined and named by linking each narrative to an appropriate theme. Finally, in the sixth phase, the narratives of the participants were presented under each generated theme in a concise manner.

### Ethical Considerations

Ethics approval was granted by the Human Research Ethics Committee (Medical) located at the University of the Witwatersrand (M190449). All participants provided written informed consent to participate and consented to have the interviews audio recorded.

Although the information provided by the participants was captured on an audio recorder, the data were protected by securely storing them in a password-protected computer in a secure locked cabinet. The identities of the participants were protected through the deidentification of all their personal information and characteristics. The transcripts contained ID numbers that represented each participant’s response.

## Results

### Quantitative Results

The sociodemographic characteristics of the participants (N=363) are presented in Table 1. Of the 363 participants, 174 (47.9%) were hard to reach telephonically, whereas 189 (52.1%) were easy to reach telephonically. Most participants (133/243, 54.7%) who were contactable did not change their mobile number. The highest percentage of mobile number changes was observed among participants who were difficult to reach, with three-quarters of the participants (12/16, 75%) being reported to have changed their mobile number ≥2 times (Multimedia Appendix 1). Although 58% (101/174) of the participants could not be reached as the attempted call went to voicemail, 29.3% (51/174) of the participants were reported to not have taken the call when the attempted call was placed, and only 2.2% (4/174) of the participants took the call but ended it (Multimedia Appendix 2).

Table 2 shows the mobile phone information of both hard-to-reach participants and contactable participants. There was a significant difference in mobile phone ownership among participants who were hard to reach and those who were contactable, as 96.8% (183/189; *P*=.03) of those who were contactable owned a personal cell phone compared with 91.4% (159/174) of those who were hard to reach. Most of the young women (both contactable and hard to reach) had a smartphone (297/342; >80% each; *P*=.02) and did not have a phone contract (324/342; >90% each). Results indicated no statistically significant differences in these variables among participants who were hard to reach and participants who were contactable.

**Table 1:** Sociodemographic characteristics of the trial participants.

Characteristics				Failed to make contact (n=174), n (%)		Contactable (n=189), n (%)		Total (N=363), n (%)		*P* value		
**Age (years)**												.19
	18-20		70 (40.2)		59 (31.2)		129 (35.5)					
	21-23		48 (27.6)		64 (33.9)		112 (30.8)					
	24-26		38 (21.8)		51 (27)		89 (24.5)					
	27-28		18 (10.3)		15 (7.9)		33 (9.1)					
**Family size**												.78
	1		8 (4.6)		13 (6.9)		21 (5.8)					
	2		13 (7.5)		17 (9)		30 (8.3)					
	3		25 (14.4)		23 (12.2)		48 (13.2)					
	4-7		95 (54.6)		105 (55.6)		200 (55.1)					
	>7		33 (19)		31 (16.4)		64 (17.6)					
**Education and socioeconomic characteristics**												.08
	**Highest level of education**											
		Primary	57 (32.8)		42 (22.3)		99 (27.3)					
		Secondary	91 (52.3)		118 (62.8)		209 (57.7)					
		Tertiary	8 (4.6)		13 (6.9)		21 (5.8)					
		Other	18 (10.3)		15 (8)		33 (9.1)					
		Missing^a^	0 (0)		1 (0.5)		1 (0.5)					
	**Vocational activity**										.07	
		Currently enrolled in higher education institution, employed, or currently looking for a job	9 (5.2)		19 (10)		28 (7.7)					
		Not enrolled in higher education institution, not employed, or not currently looking for a job	165 (94.8)		170 (89.9)		334 (92.3)					
	**Type of dwelling unit**										.43	
		House of brick or concrete block structure on a separate stand or yard or on a farm	122 (70.1)		128 (67.7)		250 (68.9)					
		House, flat, or room on your homestead	24 (13.8)		35 (18.5)		59 (16.2)					
		Informal dwelling or shack in backyard	15 (8.6)		14 (7.4)		29 (8)					
		Informal dwelling or shack not in backyard	7 (4)		3 (1.6)		10 (2.7)					
		Other	6 (3.4)		9 (4.8)		15 (4.1)					
	**Were there times in past 12 months when members of your family went hungry because there was not enough food in the house to eat**										.12	
		Yes	68 (39.1)		59 (31.2)		127 (35)					
		No	106 (60.9)		130 (68.8)		236 (65)					

^a^Missing refers to missing data or missing values that have not been stored for the education level of some participants (could be attributed to incomplete data entry or no information provided by participants).

**Table 2:** Mobile phone information of the trial participants.

Failed to make contact, n (%)				Contactable, n (%)		Total, n (%)		*P* value	
**Do you have a personal cell phone?**									*.03* ^a^
	Yes	159 (91.4)	183 (96.8)		342 (94.2)				
	No	25 (8.6)	6 (3.2)		21 (5.8)				
	Total	174 (100)	189 (100)		363 (100)				
**If yes, is it on contract?**									.20
	Yes	11 (6.9)	7 (3.8)		18 (5.3)				
	No	148 (93.1)	176 (96.2)		324 (94.7)				
	Total	159 (100)	183 (100)		342 (100)				
**Is it a smartphone?**									.32
	Yes	135 (84.9)	162 (88.5)		297 (86.8)				
	No	24 (15.1)	21 (11.5)		45 (13.2)				
	Total	159 (100)	183 (100)		342 (100)				

^a^*P*<.05 (indicates significant association between participants’ ownership of a mobile phone and contact status).

### Qualitative Results

#### Overview

Table 3 shows the sociodemographic profile of the interview participants. The mean age of the participants who were interviewed was 22 (SD 2.94) years. In addition, 50% (10/20) of the participants had a secondary school qualification, 40% (8/20) had not completed secondary education, and only 10% (2/20) had a tertiary qualification at the time of the assessment. Almost all the participants (19/20, 95%) lived with a parent or relative, whereas only 5% (1/20) of the participants lived with her partner and 65% (13/20) of the participants were not in a relationship at the time of assessment.

Textbox 1 presents an overview of the superordinate themes and subthemes generated from the qualitative analysis. As presented in Textbox 1, this section is sorted into the narrative findings, with illustrative quotations provided for each superordinate theme and subtheme.

**Table 3:** Sociodemographic profile of the interview participants.

Respondent number	Current age (years)	Highest level of education attained	Lives with	Relationship status
Participant 1	25	Secondary school qualification	Siblings and child	Single
Participant 2	22	Tertiary qualification	Parent	Single
Participant 3	21	Secondary school qualification	Parents, child, and sibling	Single
Participant 4	22	Grade 11	Parent, siblings, and child	Single
Participant 5	28	Grade 11	Parent, sibling, children, and relatives	Single
Participant 6	20	Grade 11	Parent and siblings	In a relationship
Participant 7	21	Secondary school qualification	Parent	Single
Participant 8	26	Grade 11	Partner	In a relationship
Participant 9	20	Secondary school qualification	Parent, siblings, and relatives	Single
Participant 10	24	Grade 10	Grandparent, siblings, and children	In a relationship
Participant 11	22	Grade 11	Grandparent and relatives	Single
Participant 12	24	Grade 11	Parent, siblings, and relative	In a relationship
Participant 13	20	Secondary school qualification	Parent, sibling, and child	Single
Participant 14	21	Currently doing matric	Sibling, parent, and child	In a relationship
Participant 15	25	Secondary school qualification	Relatives	Single
Participant 16	20	Secondary school qualification	Parent, siblings, and child	In a relationship
Participant 17	20	Secondary school qualification	Siblings	Single
Participant 18	22	Secondary school qualification	Parents	Single
Participant 19	22	Tertiary qualification	Grandparents and relatives	Single
Participant 20	24	Secondary school qualification	Sibling, relatives, and child	In a relationship

**Themes focusing on young women’s reasons for being hard to reach.**

**Superordinate theme and subthemes**

- Mobile phone technical issues
- Coverage issues
  Electrical unreliability
  Network connectivity issues
- Lack of ownership of personal cell phone
- Unregistered number
- Use patterns
  Cell phone theft in the community
  Stalking behavior
- Inconsistent SIM use
- Availability of data and airtime
- Lack of interest in possessing a mobile phone

#### Mobile Phone Technical Issues

Technical barriers were reported by most participants as a major challenge that affected the efficiency, functioning, and usability of their mobile phones. Key challenges reported by the participants that resulted in inherent limitations in operating their mobile phones included poor battery life, faulty charging system, mobile phone and app crashes, and an inadequate touch screen response. Such challenges resulted in the participants not being easily contactable or contactable at all. Some participants resorted to changing their mobile phones or using alternative communication methods such as using the phones of individuals within their social networks:

I’ve experienced battery challenges and the charging system, ja that’s the only thing that’s been wrong with my phone. I had to charge my phone a certain way and I couldn’t even use it until I finished charging and the battery will finish fast sometimes when I’m trying to do something.

Participant 2, 25 years old, single

The phone I was using, it lived on life support. I had to use it while it was in a charger at home. The charger also was faulty so sometimes I would think it’s on and it’s off.

Participant 9, 20 years old, single

It sometimes jams. The battery finishes quickly while you are chatting. So those are the challenges that I face with my phone.

Participant 16, 20 years old, in a relationship

My phone has problems. Like it just switches off, because it fell once, and now every time when it hits hard or something, it just switches off, and it is going to take time for it to switch on, so I wait for it to switch on.

Participant 1, 25 years old, single

The touch screen doesn’t work properly now because it fell sometime last year. I don’t know if it’s a space issue or just the phone itself because sometimes when I get incoming calls the phone rings but then I can’t touch it for me to be able to pick up the call because of the touch screen problems with the phone.

Participant 13, 20 years old, single

It has a tendency to freeze if it fell, maybe if I’m receiving a call, I can’t answer it because the phone is frozen, you know.

Participant 15, 25 years old, single

#### Coverage Issues

Coverage issues such as network and electricity made it difficult for some participants to be contactable. Participants indicated that they could not receive calls or messages or had to change their mobile SIMs because of challenges concerning the connectivity, reliability, and quality of the mobile network. This included signal weakness and network availability issues with some mobile service providers being cited as having general network unreliability or the network being offline in some instances. Participants reported experiencing unreliability of the electric supply owing to frequent power cuts. This greatly affected their ability to use their mobile phones, thus being unable to be in touch with people:

My network becomes very slow, sometimes I don’t even get calls I just get messages that I have a missed call but then it didn’t even ring if you don’t have electricity. It’s been hectic hey, we didn’t have electricity on Sunday.

Participant 2, 22 years old, single

It has been a while since we have been without electricity but right now, we don’t have it because they said that the substation was on fire.

Participant 7, 21 years old, single

I first bought the Telkom sim card but then I don’t know what happened with the coverage in Orlando, but it was giving me problems, so I couldn’t get network, so I used my MTN sim card. I think I got it like two years ago. I was not using it as frequently as the Telkom number, so I had to use the MTN number.

Participant 9, 20 years old, single

Telkom network is problematic, sometimes you want to make a call, it will tell you that there is no service, things like that. Sometimes the sim card does not connect. I will find myself taking it out and putting it back on but still, so I decided to change MTN, then, MTN gave me a problem of taking my airtime every time when I recharged.

Participant 1, 25 years old, single

#### Lack of Ownership of Personal Cell Phone

Some participants indicated that they were not easily contactable because they do not currently own their own personal cell phone or previously did not own one. The reasons attributed to the lack of ownership ranged from the phone being reported to be broken, stolen, lost, or not having one at all. Despite this, participants used alternative means of communication to try and be contactable. Participants reported using their social networks to try and solve the problem of not having a mobile phone by sharing a phone with their siblings, peers, or partners. However, sharing a phone limited their ability to stay connected with others and to be reachable because their time with the device was limited. In some cases, this was exacerbated by the participants not living close to the people they shared the phone with. In other instances, participants’ social networks would not relay the message back to them. These narratives are reflected in the following excerpts:

I didn’t have a phone for two months, so I spent quite a while without a phone. I used parents’ phones. It was for when people couldn’t get hold of me, they would call my parents for emergencies.

Participant 18, 22 years old, single

I didn’t have a phone for four months. I was not reachable. I used my boyfriend’s phone. The first number that I applied with is my grandmothers, that was the first one. I did not have a phone at that time, and so I applied with her phone, and they tried to call her, but it did not get through to her. Because her phone, she is a person that does business, it would switch off and she would not find the charger, and then it would be charged after a while, and then I gave them the number of my partner, and then they said that they can’t get me with that one as well.

Participant 10, 24 years old, in a relationship

I stayed for a long time without a phone. I think it had been two years without having a phone. I used to borrow my friend’s phone if for instance there is someone who had asked for my number, then I would give them my friend’s number, and my friend would tell me that so and so had phoned, they called me because you don’t have a phone. So, when people wanted my number, I would give them my friend’s number. It was not easy for them to get hold of me because sometimes I am at my home, and she is at her home.

Participant 1, 25 years old, single

Other participants further highlighted that they were not contactable because their mobile phone was stolen or had gone missing:

I lost my phone and then I took time to do the sim swap and by the time I decided to do it, they had already given the number to someone else.

Participant 9, 20 years old, single

I started by using said provider, yes I was using said provider first, and now I am on another provider because my phones got lost.

Participant 11, 22 years old, single

I had a phone, it was last year, I had another phone and they mugged me in March.

Participant 20, 24 years old, in a relationship

One participant lost her mobile phone due to theft, but her mobile SIM was still in her possession. As an alternative means of communication, she would insert her SIM card in other people’s phones and reach out to people given that she did not have the financial means to buy a new phone:

I was going to the mall and then I was mugged but then I have a sim card which I did a sim swap, yes so every time I need a phone, I can contact people I wasn’t able to contact.

Participant 12, 24 years old, in a relationship

#### Unregistered Number

Another factor contributing to the difficulty in reaching participants is the unregistered status of their mobile numbers. Some participants reported that they had lost their phones with the mobile SIM card. Two participants reported that they bought new phones and SIM cards and went to a mobile phone store to get a SIM swap, but the mobile phone store could not perform the SIM swap. One reason for this was because their previous mobile numbers were no longer registered under the participants’ names and had been reassigned to new owners. Another reason was the inability to recall the last 5 numbers called on the SIM, which is a requirement for performing a SIM swap. Consequently, participants had to purchase new SIM cards with new mobile numbers, different from those recorded by the trial:

I had to go this year January to Telkom to do a sim swap because I had applied for jobs with that number. Then when I got to Telkom, they told me that that number was not registered under my name, you see these sim cards that you get on the streets. So, I had to start another sim and so I started afresh with that number.

Participant 17, 20 years old, single

They took my number in January and then I got this one in February.

Participant 11, 22 years old, single

I lost the phone and then when I went to do the sim swap, they told me I couldn’t do it because the numbers were wrong, and that I wanted the last five numbers that I last called, and so I was not able to continue with the sim swap.

Participant 7, 21 years old, single

#### Use Patterns

Although many participants owned a personal cell phone, an inconsistent pattern of mobile phone use was observed in the narratives of the participants, which impacted the ability to contact them. Some participants reported that they did not carry their mobile phones outdoors and only used the device at home. However, the reasons for this behavior were not reported by the participants:

I don’t use it when I am on the road, even when I am going to town or to the mall, I usually leave it behind.

Participant 3, 21 years old, single

When I go outside, I don’t take it with me, I leave it at home. I use it when I know that I am not going anywhere.

Participant 11, 22 years old, single

Sometimes I’m at school, and sometimes I’m busy and I left it at home and went to the shops, ja I’m not on my phone every time, I just use my phone only when I do important things, ja.

Participant 14, 21 years old, in a relationship

I couldn’t go out with it, like I had to use it at home. When I go out, I leave it at home so I had to tell the person that I am meeting that we are meeting at a specific time and place, and I just had to hope they will be there.

Participant 9, 20 years old, single

Conversely, some participants reported that their communities were not safe and there was a high rate of mobile phone theft in their area. This prompted them to not use their mobile phones in public. This has contributed to participants not being easily contactable given that they use their mobile phones inconsistently:

I don’t carry my phone in the streets. It’s not safe because most of the time there’s people taking other people’s phones, most of the time, like maybe in a week we’ll hear about 2 people, 3, ja so it’s not safe.

Participant 2, 22 years old, single

I use when I am at home alone, outside there are a lot of thieves, when you are outside and you are using your phone, they take it from you.

Participant 10, 24 years old, in a relationship

Conversely, another participant indicated that she was no longer comfortable using her phone or taking calls as she had experienced continuous threatening harassment over the phone from an unknown individual. This thus resulted in other people finding it difficult to reach her:

There were people stalking me, and I didn’t know where they were getting my number from. They would tell me where I was, meanwhile, I don’t know them and I didn’t see them, then I decided to change my number.

Participant 3, 21 years old, single

#### Inconsistent SIM Use

For other participants, their inconsistent mobile phone behavior was largely because of the participants owning dual SIM cards. Participants reported that they often used one mobile number more than the other, whereas another participant indicated that she had to change her SIM cards frequently given some technical issues she was experiencing with the card. Similarly, another participant reported changing her SIM cards frequently because she would lose 1 SIM because of repeatedly changing mobile devices and thereafter had to replace it with another SIM card. Another participant’s inconsistent use of her mobile phone was largely because of her younger sibling who did not own his own personal cell phone. Given this circumstance, she shared her phone with her younger sibling as he frequently took out the participant’s SIM card and put his own for a certain period. This greatly affected the participant’s ability to receive calls or messages, thus making her seemingly unreachable. Other participants indicated that the procedures for performing a SIM swap are often offline every time they went to do one. This has led to the participants’ losing interest in doing a SIM swap and has prompted them to change their mobile number. All these factors have thus contributed greatly to the participants being unreachable:

The sim card used to get lost because I kept changing the phones. I was using the other network’s * number. The 060. So now I am using another network. I don’t know the other network’s number, but this one.

Participant 10, 24 years old, in a relationship

My younger brother likes using it a lot because he likes logging in on Facebook and chat with his friends and then bring it back. He takes out my starter pack and puts in his own. Maybe they call me, and it goes to voicemail, that is because my sim card is out.

Participant 1, 25 years old, single

I have two numbers and there is one that I always use and the other one that I use sometimes, because I have two sim cards, and both are in the phone. So sometimes I give people the number that I don’t use every day.

Participant 16, 20 years old, in a relationship

I used to use two starter packs because I wanted to use Telkom and MTN, and at the time I was using Cell C as well. I had a lot of starter packs, and I changed the numbers a lot. Telkom was giving me problems, and then MTN used to take airtime every time when I recharged. I didn’t know where my airtime was going every time when I recharged the number, so yeah, I used to change the number a lot.

Participant 1, 25 years old, in a relationship

Because when I do a sim swap, they ask a lot of questions, so I sometimes find that it is better if I change the number. Yes because this time I had to do a sim swap, and they said I should go to Shoprite with my ID and my proof of address, when I got to Shoprite, they said that their machines were offline, and then that is where I gave up, and then I said it is better that I change my numbers, because the numbers of most of the people that I know, I have them, I have written them in my book.

Participant 11, 22 years old, single

You know what, I was tired of changing number so I decided that you know what, let me stick to this number because a lot of people have this number, and they call me on it, when I give out my CVs, this is the number that I use, so I don’t take it out, this is the one that I am using.

Participant 17, 20 years old, single

Interestingly, 1 contrary finding from 1 participant’s narrative showed that the participant had the same number for >10 years and decided not to change her number to ensure that she remained contactable:

I’ve had this number for more than 10 years I think, the reason why it was more it made sense to do a sim swap instead of changing numbers it was for convenience and with this phone number I had registered it on a lot of platforms, you know when you apply online when they ask you to leave your details you leave your number, so the first thing that came to mind is that for people to be able to get hold of me it’d better to have the same number and also because had lost contacts as well for me to get back my contacts I had to have the number I was using.

Participant 15, 25 years old, single

#### Availability of Data and Airtime

Other participants reported that a major challenge that they face, which has resulted in them not being able to stay in contact with people or being difficult to reach, is that airtime and data costs are very steep, and they lack the financial ability to afford data and airtime and thus cannot purchase it regularly. As a result, there are times when they do not have any airtime or data on their phone and thus cannot make or return any calls or messages received:

When I don’t have money where I am, or I don’t have money at that time. It depends on whether I have data at the time. If in a week my data finishes on Wednesday, then I will buy it on Sunday. There are days when I don’t have data. Maybe for a short while, for maybe two or one week.

Participant 16, 20 years old, in a relationship

I was supposed to attend the interview today and I was unable to go, because I did not have data and I saw the message on a Friday.

Participant 3, 21 years old, single

Before I bought a new phone, I used Vodacom, but I just didn’t like it because their data and airtime are expensive. So, the other phone came with an MTN sim, and I carried on with that one.

Participant 18, 22 years old, single

#### Lack of Interest in Possessing a Mobile Phone

Some participants reported no interest in possessing a mobile device to stay in contact with their social networks or being contactable on the phone. One of the common reasons for this was that the participants were often in the vicinity of their social networks and could communicate in person as opposed to communicating via mobile phone. Others mentioned that they do not have friends with whom to communicate, whereas others hardly used their phones and often misplaced it given the lack of importance they give to owning a phone:

Not that important, it’s not that important in fact. Because I don’t spend a lot of time on the phone, so I wouldn’t say that it is important. Eh, they can contact me during the say but sometimes I don’t answer the phone. I think I don’t like it that much to have it all the time, I feel like I can stay without a phone.

Participant 16, 20 years old, in a relationship

It’s not that important. It’s just to keep boredom off. I don’t care much about the phone.

Participant 10, 24 years old, in a relationship

I don’t spend a lot of time on the phone, so I wouldn’t say that it is important. I think I don’t like it that much to have it all the time, I feel like I can stay without a phone.

Participant 16, 20 years old, in a relationship

I am very reckless with my phone. I don’t use my phone often so I just leave it on the table or the cupboard or wherever and then I leave the house and people will call me and not reach me until I started to realise that okay, I need to send my CV somewhere and a phone is necessary to have on you.

Participant 5, 28 years old, single

One participant indicated that she had previously lost her phone before purchasing a new one months later. The amount of time she spent without a phone resulted in her losing interest in possessing a phone as she had grown accustomed to being a nonmobile user:

I didn’t see the importance of having a phone. I stayed a long time, like I didn’t care about the phone. I stayed for a long time without it, and I didn’t feel like I didn’t have a phone. So, I didn’t see the importance of having a phone, even though I saw other people with phones, but for me, I never had that feeling of not having a phone. I had already gotten used to not having a phone.

Participant 12, 24 years old, in a relationship

In contrast to these findings, other participants indicated that possessing a mobile phone was important to them and helped them to stay in touch with their social networks, to keep abreast of any job opportunities that may arise, to apply to university or college, and to access emails and the internet. Participants indicated using their phones all the time thus suggested that they were easily contactable and reachable. These findings are reflected in the following excerpts:

It’s very important because they need to find me every time I’m needed and most things I do on the phone, so I view it most times. I use my cell phone a lot, like every day.

Participant 2, 22 years old, single

I use my phone all the time like 10 hours. When I wake up, before I go to bed, and I always provide the alternative number in case my phone is maybe low, or I have lost it. I always put in an alternative number.

Participant 9, 20 years old, single

It’s important because even the study get hold of me on that phone. When people are looking for me, they get hold of me on that phone. Even now the study helped me because if I didn’t have a phone, I would not be reachable because it has been a while since I have been here.

Participant 19, 22 years old, single

## Discussion

### Principal Findings

This study aimed to explore the challenges that participants experience in terms of being contactable through mobile phones in a trial conducted in Soweto, South Africa. This study highlighted that although most participants who are difficult to contact do own a personal cell phone, which is a smartphone, most of them face a myriad of interrelated mobile phone challenges, particularly technical and coverage issues, mobile theft in their communities, and high costs of data and airtime. Consequently, these challenges contribute to participants’ need to change their mobile numbers, which subsequently has the potential to affect their reachability. The novelty of this study was the use of a mixed methods research design. It was important to conduct a study of this nature within the context of Soweto, a resource-constrained setting, which is characterized by a short supply of free public Wi-Fi, unequal mobile coverage, and comparably high data costs [40], among other factors.

This study was descriptive in nature. Therefore, caution should be exercised when interpreting the results of this study as no significant associations were found in the quantitative analysis. The quantitative results of this study showed that a large proportion of individuals owned a mobile phone. This result is in line with previous literature, which has shown that mobile connectivity in South Africa has grown rapidly with a mobile penetration rate of >95%, with 91% of all phones being smartphones [1,17,24]. Although this was also corroborated by the qualitative findings, a key finding was that some participants were mobile phone sharers, whereas others had no phones at all and thus relied on their social networks who had phones and often depended on using these phones for a short while to be in touch with other individuals. Previous literature has shown that the use of mobile phones is an inherent part of people’s lives, which exists in socioeconomic practices and realities [15]. Although South Africa has seen an exponential increase in mobile phone ownership and access, the high cost of mobile phones still represents a formidable barrier that limits mobile phone use [40]. In fact, the lack of income has been reported as the primary factor in the lack of phone ownership in many LMICs [41,42]. This largely surpasses other probable barriers such as the perceived importance of using a mobile phone and technical literacy [41,42].

Given that this study aimed to explore the challenges that participants experience in terms of being contactable through mobile phones, overall, the quantitative and qualitative findings in this study suggest that the socioeconomic context of the participants is a major factor that has contributed to their mobile use behavior and difficulty in reaching them. Thus, the findings of this study uniquely contribute to the existing body of knowledge in various ways. First, although phone ownership has increased rapidly in South Africa, there are some important use gaps that remain pronounced, and these are evident in an LMIC such as Soweto. To illustrate this, although phone ownership may be predominantly high among both groups included in this study, their socioeconomic backgrounds are complex and create different realities, which subsequently leads to variability in mobile phone use and usability. Thus, daily realities such as unemployment, poverty, and infrastructural barriers have the potential to prevent them from fully using their devices in a sustainable manner. In addition, the challenges that were explored in this study in terms of participants being contactable through the mobile phone show that the experiences of youth are vastly different, and complexities around socioeconomic backgrounds and environmental settings create different realities pertaining to phone possession and use. Although the participants may reside in one central area, the circumstances of young people (phone sharers, phone owners, and non–phone owners) differ quite substantially, not only depending on their age, level of education, or vocational activity but also on factors such as socioeconomic circumstances, family structure, household resources, and livelihood patterns. This aligns with the evidence from the World Bank, which has shown that 50% of South African inhabitants who reside in urban settings live in low-income settings, accounting for approximately 40% of the individuals who constitute the working population and catering to approximately 60% of nonworking citizens who live in cramped family settings [29].

These previous findings align with the quantitative results of this study, which demonstrated that most participants were individuals who were still in early young adulthood (18-25 years), with >90% of them Not in Education, Employment, or Training and only 5% had a mobile phone contract. Previous studies have also indicated a digital divide along gender lines [43]. The study indicated that women are more at risk of poor mobile phone use compared with men and attributed it to economic inequality, namely, women’s lower educational attainment and poor income levels [43]. These findings illuminate the way mobile phone use is interwoven with the daily realities and experiences of the participants, which has the potential to prevent the participants from fully using their devices in a sustainable manner.

Existing research has further shown that individuals who earn a low income have limited connectivity, which is inhibited by disparities in wealth, resources, and other opportunities, presenting a challenge in terms of using mobile technologies [44]. This finding is strongly congruent with the qualitative findings, which revealed that participants faced a myriad of mobile phone challenges, leading to them being hard to reach, which is in line with what the study aimed to explore (challenges experienced in terms of being contactable over the mobile phone). Such structural inequalities that make it hard for participants to be easy to reach include coverage issues, high costs of data, and airtime and safety in their communities. Furthermore, the quantitative results of this study showed that in addition to the fact that most participants only have a secondary school qualification and are Not in Education, Employment, or Training, approximately one-third of these participants have been reported to live in households that are food insecure. Household constraints serve as important factors for people’s access to mobile technology [45]. Another study showed that ownership of a mobile phone necessitates the need to have a phone that is in good working condition (available data and airtime, functional battery, and network signal) and the financial freedom to sustain this without giving up other necessities [46]. All these factors are unevenly distributed, with those living in resource-constrained settings being the most disadvantaged [46]. These findings thus suggest that the mobile phone challenges experienced by the participants are interwoven with their socioeconomic backgrounds, thus making it difficult to maintain mobile technology use.

This study further found that the proportion of those who own phones decreased with each successive age category for both participants who were hard to reach and those who were contactable. These findings are supported by the previous literature, which showed that age is a social determinant of digital inequality [22]. A curvilinear relationship was found between age and digital use in LMICs [22]. For instance, 1 study conducted in India showed that phone ownership was >66% for participants aged 15 to 24 years but only 55% for those aged 25 to 44 years [41]. In addition, a study conducted in Tanzania found that women aged ≤30 years were 17% less likely to remain phone owners (Roessler, P, unpublished data, December 2018).

Mobile technologies have the potential to improve health behaviors among individuals participating in clinical research [38]. For instance, the COVID-19 pandemic has led to an increased need to use mobile technologies for health promotion because of social distancing and nationwide lockdowns [47]. This reliance on mobile technologies means that challenges in reaching these participants could disrupt the continuity of health promotion for both research and clinical practice. Participants could, for example, seek health advice outside of their health care system or not attend their regular health sessions [39] owing to unresolved concerns via telephone. This may thus lead to clinical interventions being highly inefficient. Thus, using mobile phones in any clinical study can inform how these devices could be used in future interventional research. Although this study found that the mobile challenges that participants face are mostly influenced by structural inequalities, it is pivotal to note that addressing and alleviating these structural inequalities is beyond the scope of this study.

However, several feasible solutions exist for the problems encountered in this study. To maximize engagement in mobile health studies, health promotion initiatives can be delivered via in-person consultations or sessions with the participants. These in-person consultations would afford participants the opportunity to have increased personal contact with the study team and to obtain study support and personalized study feedback. This strategy has proved to be successful in a previous clinical trial where personalized care, including allowing participants to share their personal problems and enabling participants to increase contact with the study team or investigators, helped in maximizing engagement [48]. This could include fieldwork to complement the use of mobile technologies and thus ensure participant retention. Furthermore, advisory groups could be created by bringing together the participants and giving them an opportunity to engage in discussions around crafting possible solutions to the mobile technology barriers that were identified in this study. In addition, automated health promotion messages could be relayed to the participants’ phones, particularly in instances where calls do go through but are not answered by participants.

### Strengths and Limitations

The strength of this study was the use of a convergent parallel mixed methods approach, which assisted in exploring the quantitative findings by relating the findings obtained from the qualitative and quantitative analyses. The use of this method thus helped in providing comprehensive insight into the mobile phone behaviors of young women and the factors that contributed to this behavior. A limitation of this study was that the quantitative analysis was only restricted to the first instance (ie, the first call attempt in which some young women were hard to reach and some were easily contactable). Therefore, repeat instances or call attempts for subsequent monthly sessions were excluded. This could have provided a broader longitudinal picture of the time from randomization to infancy of all call attempts and whether any demographic characteristics were associated with these changes. However, this was beyond the scope of this study as the focus was only on providing a descriptive profile of women who were hard to reach while comparing their demographic profile with those who were easily contactable.

### Conclusions

Despite the availability of mobile technology and network accessibility, there are substantial economic barriers that prevent young adults from fully benefitting from technology. These barriers consequently affect health promotion and behavioral changes. For individuals to remain digitally connected, multiple strategies should be used. For research, remote data collection remains an important tool in public health research and could thus serve as a hugely beneficial mechanism in connecting with participants while actively using established relationships with participants or community-based organizations to deliver health promotion and practice. With increasing phone ownership in LMICs including South Africa, greater accessibility to less expensive or free data networks or digital platforms is needed universally across South Africa to ensure more equitable access to such technologies.

## Appendix

Multimedia Appendix 1 Contact status of the trial participants by mobile number change.

Multimedia Appendix 2 Outcome of the call attempted for participants who were unreachable.

## Abbreviations

LMIC: low- and middle-income country
REDCap: Research Electronic Data Capture

## Data Availability

The data sets generated and analyzed during this study are not publicly available because the trial is still ongoing but are available from the corresponding author on reasonable request.
