# Supplementary figures and images for: Mobile Technology Use in Clinical Research Examining Challenges and Implications for Health Promotion in South Africa: Mixed Methods Study
Source: JMIR Form Res. 2024 Apr 8;8:e48144. doi: 10.2196/48144 (PMC11036187; doi:10.2196/48144)

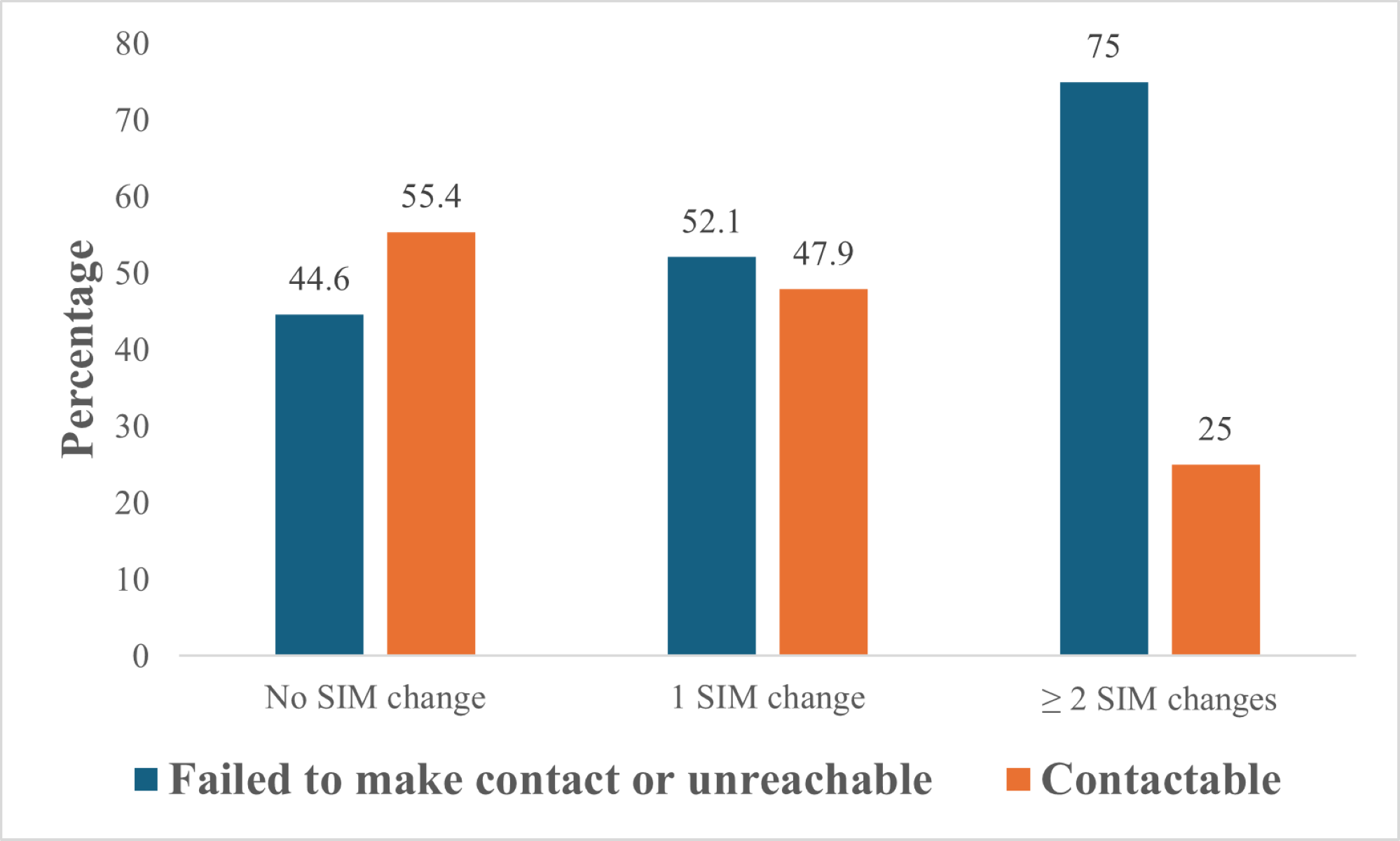

Supplement: Multimedia Appendix 1 [file formative_v8i1e48144_app1.png]

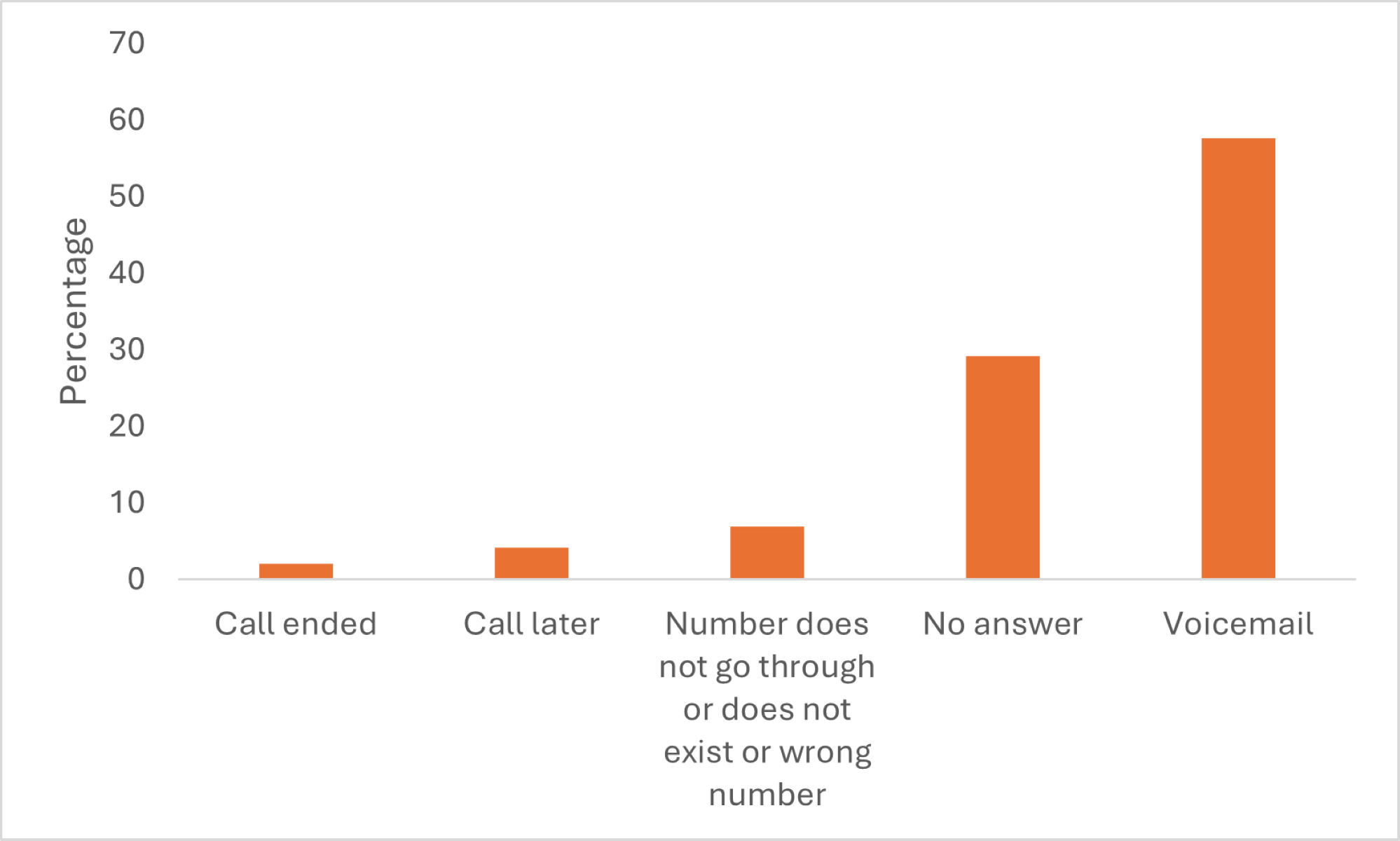

Supplement: Multimedia Appendix 2 [file formative_v8i1e48144_app2.png]
